# The Role of the Installed Base in Information Exchange Among General Practitioners in Germany: Mixed Methods Study

**DOI:** 10.2196/65241

**Published:** 2025-03-24

**Authors:** Tim Holetzek, Andreas Häusler, Kathrin Gödde, Michael Rapp, Jacob Spallek, Christine Holmberg

**Affiliations:** 1 Institute of Social Medicine and Epidemiology Brandenburg Medical School Theodor Fontane Brandenburg/Havel Germany; 2 Social and Preventive Medicine University of Potsdam Potsdam Germany; 3 Department of Public Health Brandenburg University of Technology Cottbus-Senftenberg Senftenberg Germany; 4 Lusatian Centre for Digital Public Health Brandenburg University of Technology Cottbus-Senftenberg Senftenberg Germany; 5 Faculty of Health Sciences Brandenburg Brandenburg Medical School Theodor Fontane Brandenburg/Havel Germany

**Keywords:** digitalization, general practitioners, Germany, information and communication technologies, information exchange, primary health care, digital transformation, mixed methods study, digital health, health application, qualitative interview

## Abstract

**Background:**

Digitalization is steadily advancing on a global scale, exerting a profound influence on health care systems. To facilitate acceptance of the digital transformation, guiding principles emphasize the need for digital health structures to be person-centered and promote high-quality care. This paper examines the implementation challenges within the German health care system, with a particular focus on how change initiatives engage with existing infrastructures and organizational modes of health care delivery. This approach provides a framework for analyzing how established infrastructure determines new developments while also highlighting the procedural dynamics of change and the integration of innovations within existing information infrastructures. These established infrastructures are referred to as the installed base.

**Objective:**

The aim of the study is to examine the installed base encountered by the digital transformation within the German health care system by investigating information exchange practices among general practitioners (GPs) and their communication with other health care actors.

**Methods:**

A mixed methods study including a quantitative survey and semistructured qualitative interviews was conducted. The study sample consisted of all publicly accessible GP practices (N=1348) situated in the state of Brandenburg, Germany. The survey captured demographic data, communication practices, and perceived barriers to digitalization. The interviews explored experiences with digital applications. Quantitative data were analyzed using R (R Foundation for Statistical Computing), and qualitative data were managed and analyzed in MAXQDA (VERBI Software GmbH) through content analysis.

**Results:**

A total of 250 questionnaires (response rate 18.5%) and 10 interviews with GPs were included in the analysis. GPs primarily use the telephone (n=138, 55.2%, SD 24.64), fax (n=109, 43.9%, SD 25.40), or post (n=50, 20.2%, SD 9.46) to exchange information. Newer digital communication channels such as messenger applications (n=2, 0.8%, SD 0.72) and Communication in the Medical Sector (n=1, 0.5%, SD 0.97) play a minor role. We identified three intertwined clusters displaying diverse barriers to the digitalization of GPs’ communication practices: (1) incompatibility issues and technical immaturity, (2) lack of knowledge and technical requirements, and (3) additional technical, financial, and time-related burdens. These barriers were perceived as significant deterrents to the adoption of digital tools, with older GPs more reliant on analog systems and more likely to view digitalization as a source of frustration.

**Conclusions:**

Newly established communication channels in the German health care system compete with the existing information infrastructure, which is deeply integrated into GPs’ practice routines and care processes. However, this installed base has been largely overlooked in digital transformation initiatives. While newer channels hold potential, they often malfunction and are incompatible with long-established, individualized GP workflows. Addressing these issues rather than imposing coercive measures is crucial for increasing adoption. Incorporating health care providers’ perspectives and aligning new channels with established routines can prevent frustration and facilitate a smoother digital transformation.

## Introduction

Digitalization is steadily increasing worldwide and is also massively impacting health care systems. The hope associated with digital health care systems is that they are able to mitigate the effects of staff shortages, deliver equal care in rural and deprived areas, or provide incentives for people to increase healthy living behaviors [1]. Furthermore, it is assumed that the integration of new technologies in diagnostic procedures contributes to lowering misdiagnosis rates and improving treatment processes, including the reduction of treatment delays [2]. The World Health Organization global strategy on digital health outlines principles for the digital transformation of health care systems, stating that digital health structures will be accepted when they serve quality health care and are person-centered [3]. This approach deliberately includes the health care workforce and calls for consideration of the local context, including its health care system, health needs, and resources. Simultaneously, the strategy emphasizes health data sharing and system interoperability, broadening the objectives of health care systems to encompass data production, thereby illustrating the integral role of technology in achieving these expanded goals.

This becomes particularly relevant when digitalization is considered in relation to information structures. In the context of digitalizing health care systems, the term information infrastructure describes all those “sociotechnical networks consisting of physical facilities and heterogeneous, interconnected subinfrastructures” [4] that are crucial for the exchange of information between the relevant stakeholders in a given health care system. A common way to examine processes of technological disruption in information infrastructures is to study the acceptance and adoption of new systems. Such approaches assume the presence of “old” and “new” systems, with the new either replacing or complementing the old. In this context, challenges in the transformation process are seen in organizational structures, in the users or patients, or in the technologies used [5-7]. Contrasting this perspective, Aanestad et al [8] approach implementation challenges by studying how change initiatives encounter existing infrastructures and modes of organization. Such a perspective, they argue, allows for the investigation of how existing elements shape, mobilize, and represent resources for new developments. Instead of clearly separating “old” and “new” into distinct entities, this approach allows for a focus on the procedural nature of change and the integration of innovations as part of existing information infrastructures. These information infrastructures are deeply embedded in daily work routines across organizations as well as “in social, organizational, and technical fabrics and coevolve with each other” [4,9]. Important work conducted in these routines often is tacit activities that remain hidden in formal work descriptions. Reconfiguring such work routines through technologies can lead to unanticipated and unintended consequences. Given that medical work is supported by technologies that convey information in specific formats, digitalization represents not merely a change in information transmission but actively reshapes the nature of medical practice. This is demonstrated by Vikkelsø [10] in her analysis of electronic records in Danish hospitals, showing that the introduction of digitalization leads to a reconfigured health care system rather than an improved one. She cautions us to understand the complexities and the coemergence of structure and technology rather than viewing change as a binary of good or bad, working or not working, and encourages us to conceptualize innovation in terms of the distribution of work, responsibilities, attention, and risk [10].

According to Aanestad et al [11], the successful development and implementation of information infrastructures require more than just a description of implementation objectives, necessary technological capabilities, and human skills. Instead, it is imperative to maintain a comprehensive awareness of existing organizational, institutional, regulatory, sociotechnical arrangements, established work routines, and the built environment that together form an installed base of information infrastructure in a given setting [11]. Since the existing installed base of infrastructure significantly influences the design possibilities of new infrastructure, failing to consider the existing infrastructure can lead to technical issues in newly implemented systems, poor interoperability with current systems, increased costs, and an overall lack of efficiency [12]. To accelerate the transformation process, the German federal government has enacted significant legislation in recent years to convert the German health care system into a digital one [13-20]. This includes enhancing communication among ambulatory physician offices, hospitals, and patients and resulted in mandation to implement various applications, such as digital health applications (German: *Digitale Gesundheitsanwendungen* [DHAs]) [21], electronic health records (German: *elektronische Patientenakte*) [22], or electronic medication prescriptions (German: *elektronisches Rezept*) [23]. For information exchange in the German ambulatory sector, the telematics infrastructure (TI; German: *Telematikinfrastruktur*) has been developed. The TI is a secure network specifically designed for the exchange of medical data in the German health care system. It serves as a communication platform for various stakeholders in the health care sector, such as physicians, dentists, pharmacies, hospitals, and health insurance companies. It was designed to enable the secure exchange of medical data, including patient information, electronic prescriptions, emergency data, and electronic patient records. Key technical components of the TI are so-called connectors in all health care facilities, which identify these as authorized participants using digital certificates on chip cards [23]. These connectors have had to be connected to the TI since June 2019. Relevant elements of the TI structure are electronic prescriptions, electronic certificates of incapacity, electronic patient records, but also DHAs, video communication, or the KIM (Communication in the Medical Sector, German: *Kommunikation im Medizinwesen*) module. KIM is an email-based end-to-end encrypted information transmission procedure intended to enable the secure sending and receiving of electronic physician letters and other patient-related documents between different types of providers. The use of KIM to transmit electronic certificates of incapacity for work and electronic physician’s letters has been mandatory since 2021 [16]. In the context of this paper, we define the installed base of the information infrastructure as the established communication channels that predate the current digital transformation efforts in the German health care system, which is evident in the implementation of the TI and associated channels. While a limited number of approaches address the installed base within the German context [24], the significance of specific pre-existing modes of information infrastructures for medical work has been regularly underestimated in current transformation processes to more digital information infrastructures [10]. This, in turn, has resulted in somewhat incomplete explanations for the below-average digital development in Germany compared to other European countries [23,25]. For this reason, this paper identifies practices of information exchange between general practitioners (GPs) and other stakeholders in the German health care system, using the state of Brandenburg as an example. Understanding the role and influence of the installed base of information infrastructure, we argue, will aid in anticipating challenges in the ongoing digitalization of health care services.

## Methods

### Study Design

To reach the study aim of capturing the installed base of information exchange in the health care system from the perspective of GPs, the study was set up as a mixed methods approach using a parallel design both for data collection and analysis (details on this mixed methods study following the Good Reporting of a Mixed Methods Study framework are provided as Multimedia Appendix 1). The quantitative arm of the study consisted of a survey distributed to all GPs residing in the state of Brandenburg in Germany. The qualitative arm consisted of semistructured, qualitative interviews with a subsample of GPs (10 interviews) and with citizens aged 65 years and older (21 interviews). The qualitative interviews were designed to provide an in-depth understanding of information exchange practices as well as the use and challenges of digital applications in ambulatory care settings. In contrast, the survey aimed to capture the current state of these practices, including the stakeholders involved and the methods of information exchange. Data collection for the qualitative study arm was conducted between September 2021 and October 2022, while the quantitative arm took place from March to June 2022. This paper focuses specifically on the perspective of GPs, incorporating data from both the GP survey and interviews.

### Ethical Considerations

Ethics approval for this study was obtained from the Brandenburg Medical School ethics committee (E-02-20210531), ensuring adherence to all applicable regulations governing research involving human participants such as the Declaration of Helsinki. Informed consent was obtained from all participants prior to their inclusion in the study, encompassing both quantitative and qualitative data collection and analysis, with secondary analyses explicitly permitted under the primary consent agreements. To safeguard participant confidentiality, pseudonymization was implemented for all data: IDs were assigned to participating GPs during the survey, and pseudonyms were created for each qualitative interview. Identifying information was replaced with pseudonyms in the interview transcripts. The pseudonymization lists and participants’ contact information were securely stored in paper form within a locked cabinet at the Institute of Social Medicine and Epidemiology, accessible only to authorized study personnel. All data were securely managed in compliance with institutional policies and General Data Protection Regulation guidelines. Participants were not compensated monetarily, and no identifying details appear in this manuscript or supplementary materials, ensuring both anonymity and ethical integrity throughout the research and publication process.

### Data Collection

#### Quantitative Study Arm

The questionnaire used in this study was developed by the research team in collaboration with GPs from the state of Brandenburg to align with the specific objectives of the study. The survey themes were shaped by a thorough analysis of relevant research gaps identified in the literature. Before implementation, the questionnaire was pilot-tested with 2 GPs to ensure clarity and appropriate understanding of the questions. The finalized instrument comprises 124 items and was designed to assess the communication challenges faced by GPs in their routine practice using a series of targeted queries: (1) frequency of use of specific communication channels for information exchange with specific health care stakeholders, (2) assessment of quality of exchange, and (3) expected barriers to use of digital channels (all 5-point Likert scales). In addition, sociodemographic data on age, sex, number of years of professional experience, type of practice, and population size at the practice location were collected. The questionnaire was offered as a paper-pencil or digital version. All GPs in the state of Brandenburg were invited to participate. The address data of the GPs to be invited were made available via the website of the Brandenburg Association of Statutory Health Insurance Physicians. The total of all GPs in Brandenburg was divided into 2 random subgroups, of which at baseline (T0), one group received the questionnaire by mail as a paper-pencil version including an invitation letter, study information, and consent form, whereas the other group received a mailed invitation letter to participate in a web-based questionnaire including a link and QR code. After 3 weeks (T1), a reminder in the form of postcards was sent to all GPs who had not yet participated at that time, with each postcard corresponding to an invitation for a web-based questionnaire. After 3 more weeks (T2), a second reminder was sent, with those physicians who received a paper-pencil invitation in the first wave receiving a digital invitation this time and vice versa. The web-based version was hosted on the professional survey platform SoSci Survey (SoSci Survey GmbH) under a commissioned data processing agreement to ensure secure and compliant data collection. The invitation to participate in the questionnaire was sent by post for both the paper-pencil version and the web-based version, with the invitation for the latter containing a QR code that led to the survey provider’s website. The invitation and all communication with participants were managed by the study staff at the Institute of Social Medicine and Epidemiology.

#### Qualitative Study Arm

The semistructured interviews aimed at identifying practices and routines that GPs exhibit in the exchange of information with other health care actors and how these routines affect health care delivery. From this perspective, the communication practices of GPs from the German state of Brandenburg were to be identified, which in turn should shed light on attitudes and concrete behaviors in the communication practices of GPs. In particular, the role of digitalization in medical practices was to be recorded, including the associated potentials and hurdles. An interview guideline, informed by a review of existing literature on communication practices in health care and the role of digitalization in medical practices, was developed and used for conducting the interviews (Multimedia Appendix 2). All qualitative data were collected by the first author (TH). The interviews were audio-recorded and transcribed following the methodology outlined by Kuckartz [26]. GPs were recruited for the qualitative interviews in a variety of ways. On the one hand, eligible individuals from the extended research network of the Institute of Social Medicine and Epidemiology were contacted. These were selected based on convenience sampling logic. Additionally, the General Practitioners Association Brandenburg (German: *Hausärzteverband Brandenburg e.V.*) was involved as gatekeeper, supporting the recruitment by reaching out to members informing about the study and the possibility to give an interview. Finally, interviewed GPs were asked if they knew other potentially relevant GPs who would be interested in being interviewed for the study, using a snowball sampling approach. Identified GP practices received the study information by study personnel, either via email or postal mail. GPs who provided written informed consent were included in the study.

### Analysis

#### Quantitative Study Arm

The frequency distributions of the sociodemographic data were mapped by category using a table, and relevant location parameters (mean, median, and IQR) were reported in text form. Depending on the presence of normally distributed data, 2-tailed *t* tests for independent samples or Mann-Whitney *U* tests were carried out in order to determine whether significant differences exist in the age groups according to sex. Frequency distributions for the variables were visualized using stacked bar charts. The frequency distributions for the variables were visualized with stacked bar charts, whereby categories 1 and 2 as well as 4 and 5 of the Likert scale items were combined. Spearman correlation was used to identify variations in answering behavior by age, practice site, and population size. Eta-squared statistics were calculated to determine the proportion of the variance of the variables that is explained by the grouping by practice type. Point-biserial correlations were used to examine whether participant’s sex influenced answering behavior. CIs for the Spearman correlations were calculated using bootstrap replicates (*K*=1000), and CIs for the point-biserial correlations of the sex variable were calculated using approximations by Fisher *Z*-transformations. All tests for correlations were solely exploratory. R (version 2022.07.2; R Foundation for Statistical Computing) was used for the statistical analyses. In addition to the standard R functions available in base R, the following packages were used: *readxl* (version 1.4.3), *psych* (version 2.4.1), and *boot* (1.3-28) [27-29].

#### Qualitative Study Arm

The interview materials and responses to 2 free-text questions from the survey were included in the qualitative analysis, which focused on reasons for poor exchange quality and general experiences with information exchange in general practice. The analysis was conducted using content-structuring qualitative content analysis according to Kuckartz [26]. This method aims to identify content-related aspects within the material, conceptualize the data concerning these aspects, and systematically describe them [30]. Within the framework of the analysis, deductive categories were generated from the semistructured interview guideline. During the analysis, inductive categories using open coding were added. The deductive and inductive categories were then used to categorize the free-text responses of the survey, and additional inductive categories were added to the analysis. The category construction was not theory-based and took place in several steps: first, the deductive categories for the GP interviews were discussed among the authors in terms of precision and meaningfulness. Subsequently, the entire interviews were coded using the deductive categories, and new inductive categories were determined. The newly identified inductive categories were then discussed and finalized before being applied to the interview data. In the final step, the deductive and inductive category system was used to code the free-text responses. Here, additional inductive categories were formed by the coders, then discussed and finalized, and applied to the entire free-text dataset. The analyses took place at the category level, relating the categories developed and their concrete manifestations in the material. The selection of the categories studied in more detail was informed by their importance to the research question and absolute coding frequency. Each step was carried out independently by 2 researchers. Between the individual steps, the categories developed were compared, discussed, and harmonized. All qualitative data were managed using MAXQDA 2022 software (version 22.0.1; VERBI Software GmbH).

## Results

### Sociodemographic Data

In total, questionnaires were received from 250 of the 1348 GPs contacted, corresponding to a response rate of approximately 18.5%. Of those 250 responses, 81 were collected via the web-based version, and 169 via the paper-based survey, corresponding to 32.4% and 67.6% of the total responses, respectively. Of the participants, 63.6% (n=159) were female, and the 50- to 59-year age group was particularly well represented (n=88, 35.2%). The mean age was 54.03 (SD 9.82; median 56, IQR 46-61) years. On average, the participating physicians had 19.3 (SD 12.16; median 19, IQR 8-30) years of professional work experience. Most practices were located in regions with a population density of 5000 to 20,000 or 20,000 to 100,000 inhabitants. Most participants worked in solo practices (Table 1). Age showed no significant difference between male and female participants. Thus, the distribution of age is comparable for male and female participants in this sample. A total of 10 GPs were included in the interview study, 3 of whom were male and 7 female. The participants’ ages ranged from 45 to 67 years. Of the GPs interviewed, 7 practiced in solo practices, 1 in a group practice, 1 in a joint practice, and 1 in a medical care center. The length of the GP interviews ranged from 9 to 93 minutes (mean 44.86, SD 25.1, median 46.73, IQR 28.38-52.13 minutes).

**Table 1:** Sociodemographic characteristics of survey participants (n=250): general practitioners in Brandenburg, Germany, participating in the survey study on information exchange (2022).

		Values
Age (years), mean (SD)		54.03 (9.82)
**Age group (years), n (%)**		
	30-39	22 (8.8)
	40-49	58 (23.2)
	50-59	88 (35.2)
	60-69	69 (27.6)
	70+	10 (4)
	Not specified	3 (1.2)
**Sex, n (%)**		
	Female	159 (63.6)
	Male	88 (35.2)
	Diverse	0 (0)
	Not specified	3 (1.2)
**Population size at practice location, n (%)**		
	<1000	7 (2.8)
	1001-5000	39 (15.6)
	5001-20,000	98 (39.2)
	20,001-100,000	77 (30.8)
	>100,000	23 (9.2)
	Not specified	6 (2.4)
Work experience (years), mean (SD)		19.3 (12.16)
**Work experience (years), n (%)**		
	<5	40 (16)
	5-10	31 (12.4)
	11-20	67 (26.8)
	21-30	56 (22.4)
	31-40	40 (16)
	>40	10 (4)
	Not specified	6 (2.4)
**Type of practice, n (%)**		
	Solo practice	120 (48)
	Medical care center	28 (11.2)
	Joint practice	51 (20.4)
	Group practice	29 (11.6)
	Other	17 (6.8)
	Not specified	5 (2)

### Survey

#### Frequency of Use of Specific Communication Channels for Information Exchange

**Figure 1:**
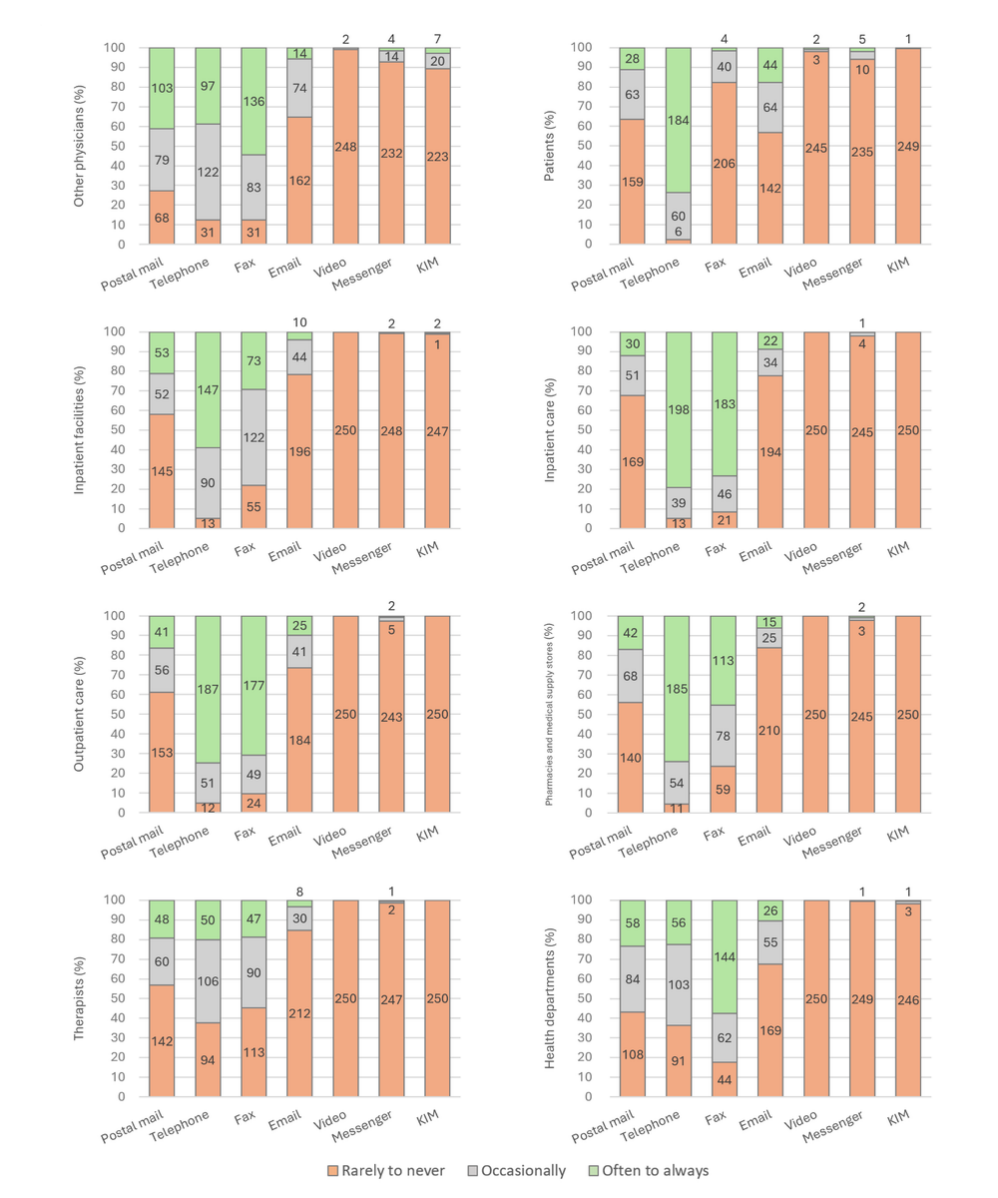
Frequency of use of selected communication channels for information exchange with various health care system actors from the perspective of general practitioners (n=250) in Brandenburg, Germany (2022). KIM: Communication in the Medical Sector.

The survey determined how often GPs exchange information with other actors in the health care system via specific channels. It was found that the exchange with all the actors surveyed takes place via channels that have been used for decades, namely, by postal mail, by telephone, and by fax. On average, GPs use the telephone (n=138, 55.2%; SD 24.64), fax (n=109, 43.9%; SD 25.40), and post (n=50, 20.2%; SD 9.46) often to always in order to exchange information with other stakeholder groups. However, the exact use characteristics of the communication channels differ depending on the communication partner. With other physicians, health departments, care actors, and pharmacies or medical supply stores, faxes in particular are sent frequently. Communication with patients is largely done by telephone and sometimes by electronic or postal mail. Email is the most frequently used of all the more digital applications (n=20, 8.2% among all communication partners; SD 4.65) but is clearly behind the older channels mentioned. Messenger applications (n=2, 0.8%; SD 0.72) and KIM (n=1, 0.5%; SD 0.97), on the other hand, are used very rarely on average (Figure 1).

Positive correlations between age or professional experience and the frequency of use of numerous different analog channels indicate that older physicians with more professional experience may be more likely to use older communication channels such as the telephone and postal mail. Email and fax, on the other hand, show negative correlation coefficients, which indicates that these communication channels are used less with increasing age. However, in all cases, the correlation coefficients are in the low range between –0.3 and 0.3 [31]. In contrast, sex, the size of the population at the practice location, and the type of practice show only minor or no correlation to the use of certain communication channels, which is why an influence on the use of the information channels surveyed is unlikely (Multimedia Appendix 3).

#### Assessment of Quality of Exchange

**Figure 2:**
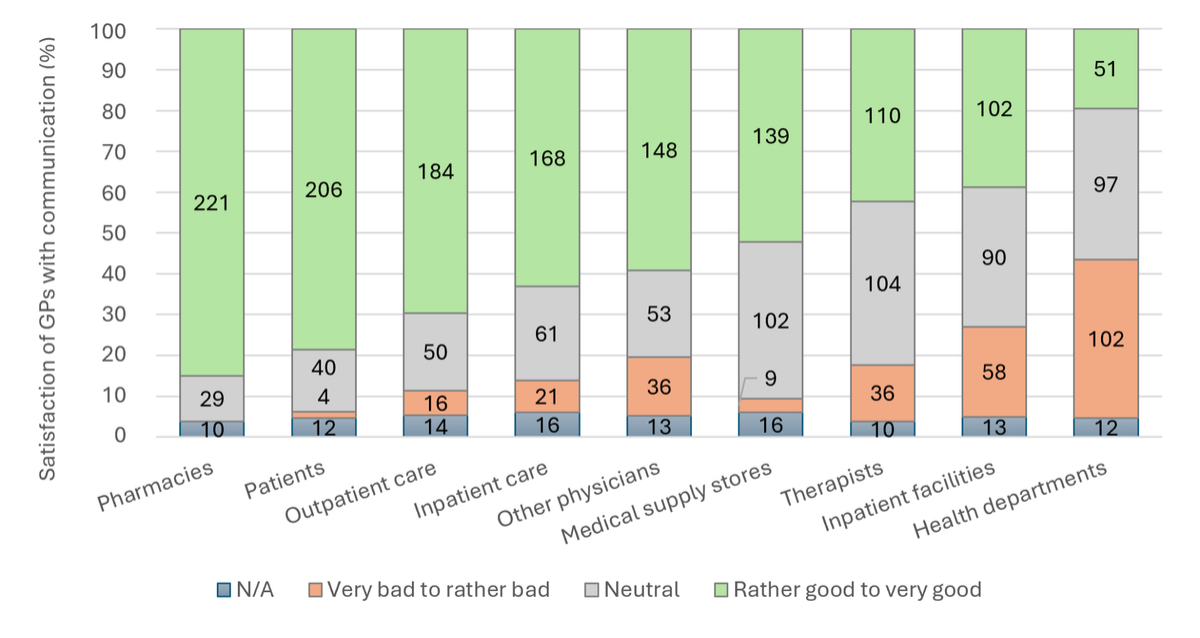
Satisfaction of general practitioners (n=250) with information exchange across various actors in the health care system in Brandenburg, Germany (2022). N/A: not applicable.

Looking at the satisfaction of the physicians surveyed with the exchange of information, it can be seen that GPs are relatively satisfied with the quality of the exchange, especially with pharmacies, patients, and care actors. Health departments, inpatient facilities, and therapists can be seen as exceptions here. Consequently, it might be argued that GPs are generally quite satisfied with the installed base of communication channels (Figure 2).

The sociodemographic variables show at best occasional links to satisfaction with the exchange of information with other actors in the German health care system. Age appears to have a certain influence on satisfaction with the exchange of information with certain actors (therapists and medical supply stores). Similarly, an increasing amount of professional years correlates with higher satisfaction with a wide range of actors (patients, health authorities, outpatient care, inpatient care, therapists, and medical supply stores). Thus, increasing age appears to contribute to greater acceptance or satisfaction with the exchange practices. All correlations are weak to moderate. Sex, population size, and practice type show little to no correlation with satisfaction with existing information exchange (Multimedia Appendix 4).

#### Expected Barriers to the Use of Digital Channels

**Figure 3:**
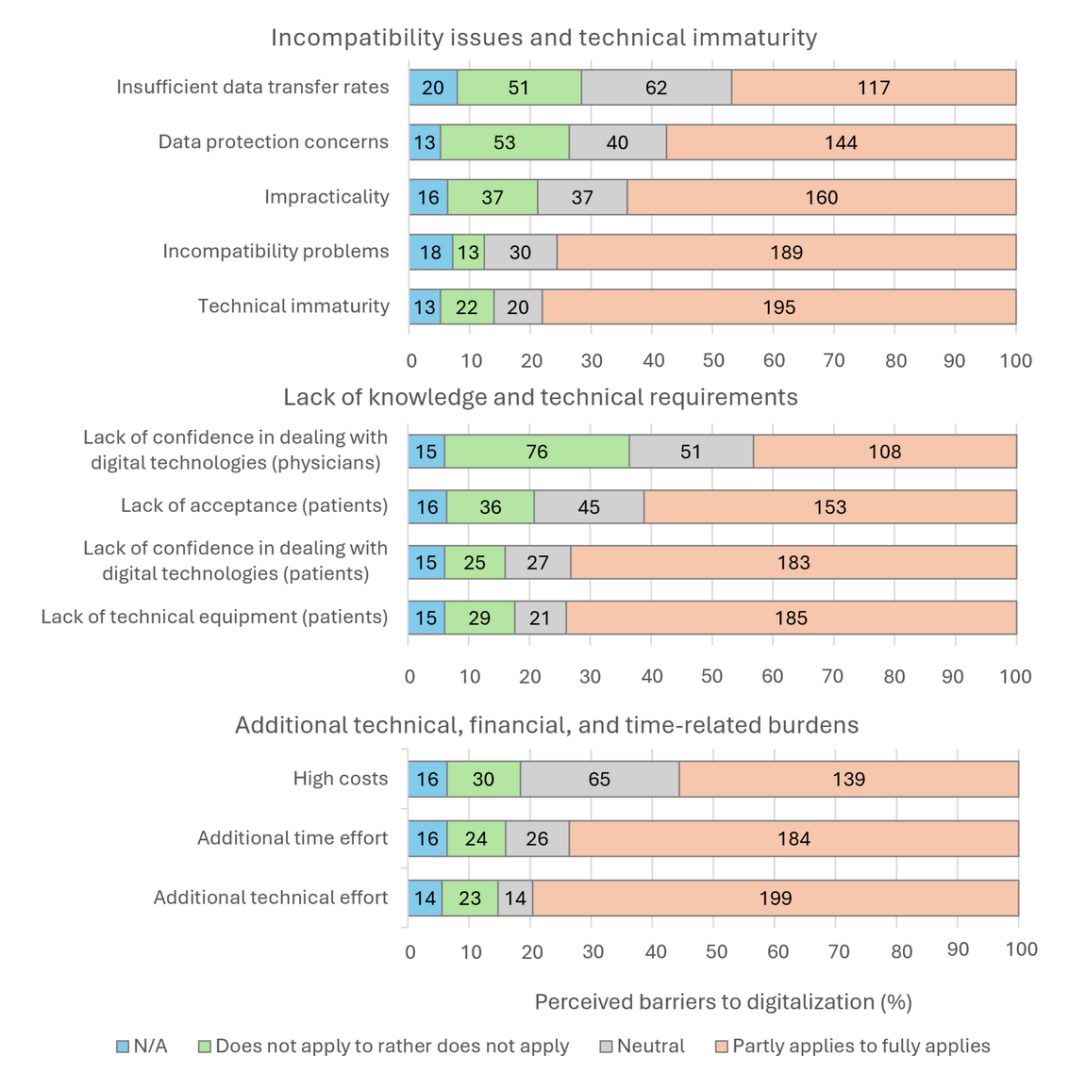
Perceived barriers to digitalization among general practitioners (n=250), categorized into 3 clusters: insights from a mixed methods study in Brandenburg, Germany (2022). N/A: not applicable.

The survey showed that GPs considered numerous barriers to digitalization to be potentially relevant to their practice (Figure 3). The barriers were assigned to 3 clusters, which are addressed again in the qualitative analysis: cluster 1—“incompatibility issues and technical immaturity” (containing “impracticality,” “technical immaturity,” “incompatibility problems,” “insufficient data transfer rates,” and “data protection concerns”), cluster 2—“lack of knowledge and technical requirements” (containing “lack of confidence in dealing with digital technologies [physicians],” “lack of confidence in dealing with digital technologies [patients],” “lack of technical equipment [patients],” and “lack of acceptance [patients]”), and cluster 3—“additional technical, financial, and time-related burdens” (containing “high costs,” “additional technical effort,” and “additional time effort”). While the additional technical and time expenditure as well as the incompatibilities and technical immaturities are particularly significant, insufficient data transfer rates and GPs’ lack of skills in using digital technologies seem less relevant.

Age appears to be relevant in the perception of numerous barriers to digitalization. With the exception of “high costs,” “data protection concerns,” and “insufficient data transfer rates,” all barriers appear to be seen as significantly more problematic with increasing age. In every case, the effect strength is low to moderate. Similar trends can be seen for the participants’ years of experience: with the exception of data protection concerns and patients’ lack of confidence using digital communication channels, all other barriers show significant correlations of weak to medium strength. Issues of high costs, data protection, and patients’ lack of confidence using digital communication channels become more pronounced, as population size increases. In some cases, there are also minor sex-related differences in the perception of digitalization barriers. Female participants are more likely to perceive data protection and insufficient data transfer rates as problematic compared to male participants. Additionally, female participants are more likely to perceive a lack of confidence in dealing with digital technologies on the side of the physician as a barrier. This might indicate a sex difference in how certain challenges or obstacles are viewed, with female participants potentially seeing these barriers as more significant or problematic. However, the effect sizes are again small to medium (Multimedia Appendix 5).

### Qualitative Results

#### Overview

While the survey was able to show to what degree the established base of communication channels is used by GPs and that certain barriers exist in the use of more recent applications, it was not yet possible to understand how these barriers manifest in everyday practice. To do so, the qualitative analyses can offer valuable insights and identify specific needs regarding digitalization. The results are arranged according to the 3 clusters mentioned earlier, which were developed as part of the categorization of the qualitative data material. They appear to be strongly interrelated.

#### Cluster 1—Incompatibility Issues and Technical Immaturity

Both the qualitative interviews as well as the free-text analyses revealed that malfunctioning technology is a particularly relevant problem in the digital exchange of information. This includes practice management software and modules of the TI, which sometimes run very slowly. In addition, there are noticeable incompatibilities between numerous existing practice management software options and the TI, resulting in frequent system crashes. This negatively affects the user experience:

I have found many colleagues to be fundamentally open to digitalization—but there is a great deal of dissatisfaction with the implementation. The devices and software are often incompatible, it takes more time [to use them], there are system crashes and registrations are complex. Instead of reducing bureaucracy, the introduction of digital structures results in printouts and digital dispatch—which makes twice as much work.

In particular, the use of KIM, which is intended to enable the secure sending and receiving of electronic physician letters and other patient-related documents between different types of providers, has been prone to numerous errors in the past and is also relatively unintuitive from a physician’s perspective and involves too many clicks on the part of the sender. In addition, faulty health insurance card readers regularly cause practice management systems to fail. The various and regularly occurring technical difficulties result in the need to consult external IT support service providers. Participating GPs had varying levels of experience with such providers, but it was frequently noted that services were unsatisfactory, often caused by the associated high costs for GPs as well as poor availability of services due to high levels of capacity use.

#### Cluster 2—Lack of Knowledge and Technical Requirements

Another barrier associated with digitalization is the lack of knowledge about technical processes, which limits the use of digital applications. This applies to older physicians or those who have little know-how in terms of digitalization:

I belong to a generation that never received an introduction to data technology. As a result, all technical applications are all Greek to me. [...] At almost 63 years old, technology is also a huge burden for me, and I don’t feel I have the time to cope with the innovations alongside my medical work.

This also affects patients, especially older ones:

Due to the increasing aging of the population, patients are increasingly experiencing cognitive problems, explanations have to be retrieved more frequently, and additional limitations (such as hearing loss) make this even more difficult. The use of digital media overwhelms almost all patients older than 80, many no longer use cell phones, PCs or tablets. In my experience, the intended extensive digitalization does not reach about 25% of my patients, and about 50% of patients with a permanent medication.

In addition to a lack of technical affinity, affected individuals, especially older patients, lack technical equipment. This includes hardware such as terminal devices (computers, smartphones, and tablets) but also an inadequate internet connection. Especially some rural medical practices only have a poorly developed internet connection at the practice location, which limits the use of the TI:

The internet connection is unfortunately inadequate, in some cases only 0.1 Mbit/s when downloading. This is a major hurdle with regard to digitalization, especially when using electronic certificates of incapacity or e-prescriptions.

#### Cluster 3—Additional Technical, Financial, and Time-Related Burdens

As a direct consequence of the previously mentioned deficits in digital information exchange, these cause enormous additional time and administrative expenses. The need for frequent error management and software updates ties up physicians’ time and limits patient care:

We are witnessing terrible changes with the introduction of the telematics infrastructure! It takes an extreme amount of extra time to personally create all the prerequisites for this. You need time for the installation, the constant updates, there is a constant need for remote maintenance for normal working functions of the practice management system. [...] A lot more “clicks” are required for every work step. I don’t see any improvement or simplification through the TI, but an extreme time burden with bureaucratic and technical tasks.

In addition, the various susceptibilities to failure of existing hardware and software result in a technology dependency, which also manifests itself in continuous error management and thus ties up time that could be used for patient care:

And it’s also a huge nuisance in everyday practice when things don’t work. If the system fails, care is actually no longer possible. You can’t do anything anymore. You can talk to the patient, but you can’t write a prescription. You can’t call up a single form. In fact, nothing is possible if the telematics infrastructure is blocked. Because it is also not possible to read the health insurance card.

The high speed of innovation, expressed in a constant stream of new applications and updates, also makes it necessary for physicians to regularly spend part of their working time keeping up to date with the latest applications and initiating their implementation in their own practices:

It takes 3-9 months between being informed about innovations and ordering the components and delivery or installation. After installation, you have to start reading up again, as you have forgotten the information in the meantime and there were five other innovations in parallel—it never stops and you can’t keep up.

Another significant negative factor is the financial outlay that digitalization entails for GPs. This includes the comparatively high acquisition and setup costs but also the maintenance and update costs, which are correspondingly high if problems occur regularly. In particular, physicians without a deeper understanding of IT and thus problem-solving skills are at risk of having to pay more money for more frequent IT services. This is likely to include older physicians in particular. Furthermore, switching practice management software providers is very costly, since the data must be transferred by external IT companies.

The perceived additional burden of the aspects described here can become problematic for a health care system, especially if it leads to physicians resigning in the face of the hurdles posed by digitalization. In this context, especially older medical practices are at risk, if owners do not feel up to the demands of digitalization. As a result, physicians may give up their professional activities prematurely, which can lead to supply bottlenecks that may not be able to be closed quickly. This is particularly true in very rural regions with a lower physician density:

If my daughter hadn’t said, with the pandemic and with all this digitalization, “I’m taking over the practice,” then it would have been a reason for me to give up the practice. Yes, it would have been a reason for me. I have also had practice nurses who are also my age, that is, those who are about to retire. And I have to tell you, that was really a big challenge for us to handle. And there I would have said, “No, I’m not going to do that to myself now at my age.” You know, first and foremost, I’m a doctor and not somebody who’s constantly dealing with technology here. Because I can see it. When I come here, there’s always some problem. There’s always something wrong with all the software and I don’t know what all. And I wouldn’t have had the nerve to do it anymore. I would have said, “That’s enough. I’m not going to do this to myself anymore.” So that’s where I’m at.

However, the barriers and especially the high bureaucratic hurdles can also have a deterrent effect on younger generations of physicians:

It’s just awful that there is such extensive interference with professional self-employment of physicians. I am not surprised if I do not find a successor for my practice. I would never become self-employed again under these circumstances, and I will close my practice at some point, not just at the age of 67—I am used up, exhausted, at the end of my tether.

## Discussion

### Principal Findings

This paper examined the role of the installed base in information exchange between GPs and other health care actors in Brandenburg, as an exemplary more rural region in Germany. The analysis revealed several relevant findings. At the time of data collection, the participating GPs very frequently interacted via communication channels that had existed for several decades, such as telephone, fax, or postal mail. The older age of physicians appears to be associated with a greater use of more traditional communication channels within the installed base and to greater skepticism toward digitalization. This is shown in the positive and significant correlations between age or professional experience and the frequency of use of numerous different older channels, indicating that older physicians with more professional experience may be more likely to use older communication channels such as the telephone and postal mail. These older communication channels work well from the perspective of those GPs’ who took part in the survey. In contrast, new communication applications developed and introduced in recent years in the context of digitalization have not been used as frequently. Among those applications, the low rate of use of the KIM service is particularly notable since KIM has been mandatory for GPs to use for information transfer while this survey was conducted. The installed base in the form of postal letters, fax, and other channels appears to compete with the newly developed KIM channel, limiting its use. Although the exact use practices varied depending on the communication partner, this general trend applies equally to all of the surveyed actors, namely, other physicians, patients, inpatient facilities, in- and outpatient care actors, therapists, pharmacies, and medical supply stores as well as health departments. As was shown, the participating GPs perceived hurdles in the use of digital communication channels, which are arranged around the 3 clusters “incompatibility issues and technical immaturity,” “lack of knowledge and technical requirements,” and “additional technical, financial, and time-related burdens.” Taken together, the hurdles perceived by GPs could result in digital channels being seen more as an additional burden than an added value, which could limit their use and ultimately jeopardize health care provision.

The results obtained here coincide to a certain extent with those of similar studies. While the user behavior of GPs toward existing information infrastructure for the German context has scarcely been examined from the point of view of the installed base, findings do exist on the barriers to the use of digital exchange channels. In a previous study, Schendzielorz et al [32] found that in a more rural area in Germany, the high cost of applications, technical problems (slow internet connection and nonfunctioning applications), the time required to process digital and analog documents, and a lack of personal contact in particular were obstacles to the use of digital technologies.

According to the “PraxisBarometer” of the German National Association of Statutory Health Insurance Physicians 2021, GPs in Germany have been noted for their rather negative attitudes toward digitalization, with technical hurdles in the use of associated applications appearing to be particularly significant, as expressed by a lack of user-friendliness, high conversion costs (financial costs, information and training requirements, and time needed), an unfavorable cost-benefit ratio, and concerns about data protection [33]. A lack of knowledge in the use of digital applications as well as a low tolerance for telemedicine and an advanced age are also not conducive to the willingness to use digital applications [34]. It was further shown that physicians rarely receive diagnostic data, doctor’s letters, or radiological findings in digital form, and just under a quarter of medical practices do not receive any digital data from other outpatient facilities. In addition, about two-thirds of medical practices did not send any treatment-relevant data in digital form themselves. Further, the frequency of errors in the use of relevant applications has increased over the past few years [33]. These trends have continued across Germany in the years following the data collection for this paper. Despite increasing use rates of corresponding applications, digitalization has not yet become fully established in practices [35,36]. The barriers seem to be similar across applications. With regard to DHAs, for example, it is regularly noted that GPs lack confidence in their ability to introduce patients to DHAs and support their use [37]. In addition, privacy, security, and legal concerns as well as costs related to reimbursement and fees are among the most stated barriers to a more intense use of DHAs [38]. Overall, various barriers to the implementation of digital applications were identified. However, these are by no means unique to Germany but are relevant in many other countries and specialist areas outside of GP care and in relation to various digital tools [39-42].

Ultimately, it can be concluded that the newly established communication channels and applications associated with the TI compete with the installed base of information infrastructure. Some GPs have been using these established channels for decades, with their practice routines and care processes built around them. These systems function adequately for many providers at a procedural level. Moreover, the newer applications experience various malfunctions that exceed the limitations of the older communication channels. If the technical problems of the newer communication channels are sustainably eliminated, the willingness of GPs to rely more on them in their practices would very likely increase. However, if the technical issues persist, health care will be severely impacted, testing the willingness and patience of the physician community. Under these circumstances, the potential of digitalization for health care can only be guessed at. To this end, adjustments must be made above all at the individual, that is, the practice level. This is all the more relevant, as the Digital Act has declared the electronic patient file as an opt-out version for 2025, which will lead to GPs in particular having to deal with the topic and technology more intensively [19].

### Strengths and Limitations

This paper is accompanied by some limitations. For example, the survey’s response rate of 18.5% (n=250) among Brandenburg GPs may come along with several limitations that warrant consideration. First, the relatively low response rate raises concerns about the representativeness of the sample, which may not adequately reflect the broader population of GPs in the region. This could limit the generalizability of the findings to all Brandenburg GPs. Additionally, nonresponse bias might be present if the GPs who chose to participate differ systematically from those who did not, potentially affecting the validity of the results. It is also possible that those who responded were more likely to have strong opinions or experiences related to the survey topic, which could skew the results. The same logic applies with regard to the interviews. Accordingly, the extent to which the results are applicable to the rest of Germany beyond the federal state of Brandenburg must be carefully considered. However, the participating physicians adequately reflected the German age structure of GPs (German: *Allgemeinmedizin* and *Interne Medizin mit hausärztlicher Tätigkeit*), according to which 7.5% (n=2561) and 8.6% (n=1478) are younger than 40 years of age, 20.7% (n=7127) and 28.5% (n=4893) between 40 and 49 years, 35.3% (n=12,153) and 37% (n=6361) between 50 and 59 years, 20.6% (n=7097) and 15% (n=2584) between 60 and 65 years, and 15.9% (n=5455) and 10.9% (n=1875) older than 65 years. The national average age is 55.3 years [43]. The sex distribution roughly corresponded to the Brandenburg GP group (n=731, 62.9% of Brandenburg GPs are female) [44]. For data protection reasons, the geographical location of the participating practices was not determined. A notable limitation of the parallel mixed methods design was the inability to iteratively refine data collection tools based on preliminary findings from either method. The concurrent collection of quantitative survey data and qualitative interviews precluded adjustments to the survey instrument to incorporate emerging insights from the qualitative interviews or modifications to the interview guide to explore trends identified in the survey responses. This limitation may have constrained the ability to capture more nuanced interconnections between the 2 datasets and to fully investigate unanticipated findings. Finally, while the results presented here show how GPs in Brandenburg communicate with other actors and what kinds of barriers they experience with regard to digitalization, they only show part of the practices in communication. Thus, the perspectives of GPs only provide a limited view of communication. Including the perspectives of GPs’ direct communication partners may do justice to the complexity of the German health care system by identifying cross-actor problems and offering more comprehensive solutions. This explicitly includes the concrete needs of patients and citizens with regard to digital communication and their views with regard to the installed base.

### Conclusions

In the exchange of information, it appears that a number of conditions must be met in order to guarantee the added value of digitalization compared to those exchange formats associated with the installed base from the physician’s point of view. To successfully establish digital communication in the long term, patience and technical support should be granted by the political leadership instead of maintaining formal coercive liability and sanctions. Innovations in information exchange should be integrated in a planned, careful, and step-by-step manner, rather than simply being implemented, to reduce the risk of undesirable effects and potential failures [45,46]. From our data, we see that the sole legislative implementation of an infrastructure component (like KIM) does not automatically result in its integration as a functional component of the installed base. Quite the opposite, it may even have counterproductive effects on resources. Considering these aspects, irritation, frustration, and even resignation could be limited. This implies that new applications should not be rolled out until their functionality is guaranteed, for example, through completed test phases with the help of pilot practices. Furthermore, the perspectives of the health care providers who use the infrastructure to be replaced should be more integrated into the planning of the adjustments [47], with particular attention to individual practice processes. Primary care in Germany is typically very individualized, with communication practices often based on decades-old routines. Digitalization is forcing such individuals, first, to adapt their established practices and routines and, second, to deal with technologies that might be completely alien to them. The frustration that arises here is reasonable and, if it translates into resignation and abandonment of practice, could potentially lead to problems and bottlenecks in care. This should be prevented. GPs play a crucial role in the digitalization of the German health care system. This is due to several factors, including their involvement in a significant portion of patient-related communication, their role in prescribing DHAs [37], and their responsibility for filling electronic health records with data [22]. Facilitating the associated processes for them seems expedient to the overall objective. In the necessary parallel existence of long-established communication channels like postal mail or fax and newer channels such as KIM, arguments need to be found in favor of the latter that do not run counter to individual practice routines.

As has been shown in this paper, especially physicians of older age and with long-established practices need to be given mediating or organizing instances to overcome the individual hurdles of digitalization. This can be done by younger, more technology-savvy colleagues, specially trained practice personnel who take on the digitalization, or other actors or bodies. In this context, a guidance concept is conceivable, where external personnel provide in-house support in primary care practices or comparable settings. Comprehensive continuing education and training programs appear to be necessary, as well as the visualization of links to existing offers. In addition, central quality criteria for training courses should be established in order to provide interested parties with guidance in selecting suitable programs. Aspects of digitalization should also be integrated into the curriculum of medical students. It seems important to identify the specific technical knowledge physicians need to effectively perform their duties.

Finally, it is relevant to remember that barriers to the use of digital communication channels can have different causes. On the one hand, technologies may not operate appropriately because the technology is prone to failure, because users do not possess relevant knowledge, or for other reasons. On the other hand, however, new applications can disrupt the general work routines of GPs, some of which may have been in place for decades and which can be assumed to have meaning and to meet the individual needs of GPs. At this point, the significance of the installed base of communication channels for health care becomes evident beyond their purely technical-material presence. Understanding how communication channels and technologies interact with practice routines can help align newer applications with the needs and demands of GPs, making them more attractive to them. In this way, health care areas that are characterized by a low provider density and a high average age of the population and medical practitioners can benefit in particular.

## Appendix

Multimedia Appendix 1 Good Reporting of a Mixed Methods Study.

Multimedia Appendix 2 Overview of themes and subthemes explored in qualitative, semistructured interviews conducted with general practitioners in Brandenburg, Germany (2021-2022).

Multimedia Appendix 3 Correlations between frequency of communication channel use in information exchange by general practitioners and sociodemographic factors (age, sex, size of population at practice location, type of practice, and years of practice experience): significant survey results from Brandenburg, Germany (2022), including correlations with communication partners such as pharmacies and medical supply stores, sorted by sociodemographic variable and strength of correlation.

Multimedia Appendix 4 Correlations between satisfaction with communication with health care system actors and sociodemographic factors (age, sex, size of population at practice location, type of practice, and years of practice experience): significant survey results from general practitioners in Brandenburg, Germany (2022), sorted by sociodemographic variable and strength of correlation.

Multimedia Appendix 5 Correlations between perceived barriers to digital communication and sociodemographic factors (age, sex, size of population at practice location, type of practice, and years of practice experience): significant survey results from general practitioners in Brandenburg, Germany (2022), sorted by sociodemographic variable and strength of correlation.

## Abbreviations

DHA: digital health application
GP: general practitioner
KIM: Communication in the Medical Sector
TI: telematics infrastructure

## Data Availability

The datasets generated or analyzed during this study are not publicly available due to data protection concerns and the written informed consent but are available from the corresponding author on reasonable request.
