# Supplementary material for: The Role of the Installed Base in Information Exchange Among General Practitioners in Germany: Mixed Methods Study
Source: J Med Internet Res. 2025 Mar 24;27:e65241. doi: 10.2196/65241 (PMC11976167; doi:10.2196/65241)
Supplement: Multimedia Appendix 1 [file jmir_v27i1e65241_app1.pdf]

Good Reporting of A Mixed Methods Study (GRAMMS) according to O'cathain A, Murphy E, Nicholl J. The Quality of Mixed Methods Studies in Health Services Research. *Journal of Health Services Research & Policy*. 2007; 13(2). doi.org/10.1258/jhsrp.2007.007074

(1) Describe the justification for using a mixed methods approach to the research question.

In the section "Methods – Study Design" we describe why a mixed-methods approach is significant for answering the research question:

To reach the study aim of capturing the installed base of information exchange in the health care system from the perspective of GPs, the study was set up as a mixed methods approach using a parallel design both for data collection and analysis (details on this mixed methods study following the GRAMMS framework are provided as Multimedia Appendix 1). The quantitative arm of the study consisted of a survey distributed to all GPs residing in the state of Brandenburg in Germany. The qualitative arm consisted of semistructured, qualitative interviews with a subsample of GPs (10 interviews) and with citizens aged 65 years and older (21 interviews). The qualitative interviews were designed to provide an in-depth understanding of information exchange practices as well as the use and challenges of digital applications in ambulatory care settings. In contrast, the survey aimed to capture the current state of these practices, including the stakeholders involved and the methods of information exchange.

(2) Describe the design in terms of the purpose, priority and sequence of methods.

The study employed a parallel mixed-methods design, where both the quantitative survey and qualitative interviews were conducted simultaneously. This design allowed for the independent collection of complementary data: the survey provided a broad quantitative overview of general practitioners' communication practices and barriers to digitalization, while the interviews offered in-depth qualitative insights into the practical challenges faced by GPs and their integration of digital tools. Equal priority was given to both methods, and the parallel design ensured that data from one method did not influence the collection of data in the other. Integration of the findings occurred during the analysis phase, where quantitative trends were contextualized with qualitative data to develop a nuanced understanding of the research problem. We have described the design accordingly on in the section "Methods – Study Design" of the manuscript.

(3) Describe each method in terms of sampling, data collection and analysis.

Information on this topic can be found in the sections "Data Collection - Quantitative Study Arm" and "Analysis – Quantitative Study Arm" as well as sections "Data Collection - Qualitative Study Arm" and "Analysis – Qualitative Study Arm":

Data Collection – Quantitative Study Arm:

The questionnaire used in this study was developed by the research team in collaboration with GPs from the state of Brandenburg to align with the specific objectives of the study. The survey themes were shaped by a thorough analysis of relevant research gaps identified in

the literature. Before implementation, the questionnaire was pilot-tested with 2 GPs to ensure clarity and appropriate understanding of the questions. The finalized instrument comprises 124 items and was designed to assess the communication challenges faced by GPs in their routine practice using a series of targeted queries: (1) frequency of use of specific communication channels for information exchange with specific health care stakeholders, (2) assessment of quality of exchange, and (3) expected barriers to use of digital channels (all 5-point Likert scales). In addition, sociodemographic data on age, sex, number of years of professional experience, type of practice, and population size at the practice location were collected. The questionnaire was offered as a paper-pencil or digital version. All GPs in the state of Brandenburg were invited to participate. The address data of the GPs to be invited were made available via the website of the Brandenburg Association of Statutory Health Insurance Physicians. The total of all GPs in Brandenburg was divided into 2 random subgroups, of which at baseline (T0), one group received the questionnaire by mail as a paper-pencil version including an invitation letter, study information, and consent form, whereas the other group received a mailed invitation letter to participate in an web-based questionnaire including a link and QR code. After 3 weeks (T1), a reminder in the form of postcards was sent to all GPs who had not yet participated at that time, with each postcard corresponding to an invitation for an web-based questionnaire. After 3 more weeks (T2), a second reminder was sent, with those physicians who received a paper-pencil invitation in the first wave receiving an digital invitation this time and vice versa. The web-based version was hosted on the professional survey platform SoSci Survey (SoSci Survey GmbH) under a commissioned data processing agreement to ensure secure and compliant data collection. The invitation to participate in the questionnaire was sent by post for both the paper-pencil version and the web-based version, with the invitation for the latter containing a QR code that led to the survey provider's website. The invitation and all communication with participants were managed by the study staff at the Institute of Social Medicine and Epidemiology.

Analysis – Quantitative Study Arm:

The frequency distributions of the sociodemographic data were mapped by category using a table, and relevant location parameters (mean, median, quartiles) were reported in text form. Depending on the presence of normally distributed data, 2-tailed t tests for independent samples or Mann-Whitney U tests were carried out in order to determine whether significant differences exist in the age groups according to sex. Frequency distributions for the variables were visualized using stacked bar charts. The frequency distributions for the variables were visualized with stacked bar charts, whereby categories 1 and 2 as well as 4 and 5 of the Likert scale items were combined. Spearman correlation was used to identify variations in answering behavior by age, practice site, and population size. Eta-squared statistics were calculated to determine the proportion of the variance of the variables that is explained by the grouping by practice type. Point-biserial correlations were used to examine whether participant's sex influenced answering behavior. CIs for the Spearman correlations were calculated using bootstrap replicates (K=1000), and CIs for the point-biserial correlations of the sex variable were calculated using approximations by Fisher Z-transformations. All tests for correlations were solely exploratory. R (version 2022.07.2; R Foundation for Statistical Computing) was used for the statistical analyses. In

addition to the standard R functions available in base R, the following packages were used: readxl (version 1.4.3), psych (version 2.4.1), and boot (1.3-28) [27-29].

Data Collection – Qualitative Study Arm:

The semistructured interviews aimed at identifying practices and routines that GPs exhibit in the exchange of information with other health care actors and how these routines affect health care delivery. From this perspective, communication practices of GPs from the German state of Brandenburg were to be identified, which in turn should shed light on attitudes and concrete behaviors in communication practices of GPs. In particular, the role of digitalization in medical practices was to be recorded, including the associated potentials and hurdles. An interview guideline, informed by a review of existing literature on communication practices in health care and the role of digitalization in medical practices, was developed and used for conducting the interviews (Multimedia Appendix 2). All qualitative data were collected by Tim Holetzek. The interviews were audio-recorded and transcribed following the methodology outlined by Kuckartz [26]. GPs were recruited for the qualitative interviews in a variety of ways. On the one hand, eligible individuals from the extended research network of the Institute of Social Medicine and Epidemiology were contacted. These were selected based on convenience sampling logics. Additionally, the General Practitioners Association Brandenburg (German: Hausärzteverband Brandenburg e.V.) was involved as gatekeeper, supporting the recruitment by reaching out to members informing about the study and the possibility to give an interview. Finally, interviewed GPs were asked if they knew other potentially relevant GPs who would be interested in being interviewed for the study, using a snowball sampling approach. Identified GP practices received the study information by study personnel, either via email or postal mail. GPs who provided written informed consent were included in the study.

Analysis – Qualitative Study Arm:

The interview materials and responses to 2 free-text questions from the survey were included in the qualitative analysis, which focused on reasons for poor exchange quality general experiences with information exchange in general practice. The analysis was conducted using content-structuring qualitative content analysis according to Kuckartz [26]. This method aims to identify content-related aspects within the material, conceptualize the data concerning these aspects, and systematically describe them [30]. Within the framework of the analysis, deductive categories were generated from the semistructured interview guideline. During the analysis, inductive categories using open coding were added. The deductive and inductive categories were then used to categorize the free-text responses of the survey, and additional inductive categories were added to the analysis. The category construction was not theory-based and took place in several steps: first, the deductive categories for the GP interviews were discussed among the authors in terms of precision and meaningfulness. Subsequently, the entire interviews were coded using the deductive categories, and new inductive categories were determined. The newly identified inductive categories were then discussed and finalized before being applied to the interview data. In the final step, the deductive and inductive category system was used to code the free-text responses. Here, additional inductive categories were formed by the coders, then discussed and finalized, and applied to the entire free-text dataset. The analyses took place at the

category level, relating the categories developed and their concrete manifestations in the material. The selection of the categories studied in more detail was informed by their importance to the research question and absolute coding frequency. Each step was carried out independently by 2 researchers. Between the individual steps, the categories developed were compared, discussed, and harmonized. All qualitative data were managed using MAXQDA 2022 software (version 22.0.1; VERBI Software GmbH).

(4) Describe where integration has occurred, how it has occurred and who has participated in it.

Integration occurred during the analysis and interpretation phases. Survey results provided a broad overview of communication practices and barriers, while qualitative data enriched these findings by highlighting individual barriers, technical issues, and patient-related challenges. Both data types were synthesized to identify overarching themes, such as the dominance of the *installed base* of communication channels and barriers to digital adoption. The research team collaborated in interpreting the integrated results to ensure consistency. In the text, the integration of the data becomes apparent particularly from the section “Qualitative Results”.

(5) Describe any limitation of one method associated with the present of the other method.

We report one limitation, namely that parallel data collection leads to the fact that the survey tools used cannot be informed retrospectively by results already obtained (section Strengths and Limitations):

A notable limitation of the parallel mixed methods design was the inability to iteratively refine data collection tools based on preliminary findings from either method. The concurrent collection of quantitative survey data and qualitative interviews precluded adjustments to the survey instrument to incorporate emerging insights from the qualitative interviews or modifications to the interview guide to explore trends identified in the survey responses. This limitation may have constrained the ability to capture more nuanced interconnections between the 2 datasets and to fully investigate unanticipated findings.

(6) Describe any insights gained from mixing or integrating methods.

The integration of quantitative and qualitative methods provided a more comprehensive understanding of the research problem than either method alone could have achieved. While the survey data quantified the reliance on traditional communication methods and the frequency of perceived barriers, the qualitative interviews uncovered contextual nuances, such as the frustrations associated with system incompatibility and the administrative burden of digital tools. This combination revealed the entrenched role of the *installed bas*e in shaping communication practices and the multifaceted nature of barriers to digitalization. We illustrate this in both the Results section and the Discussion.
