# Supplementary material for: The Role of the Installed Base in Information Exchange Among General Practitioners in Germany: Mixed Methods Study
Source: J Med Internet Res. 2025 Mar 24;27:e65241. doi: 10.2196/65241 (PMC11976167; doi:10.2196/65241)
Supplement: Multimedia Appendix 2 [file jmir_v27i1e65241_app2.docx]

| **Themes** | **Subthemes** |
| --- | --- |
| Information provision to patients | - Channels and methods of information exchange before and after the consultation session (means of communication, frequency, purpose, and timing) |
| Information exchange with other physicians | - Channels and methods of information exchange with other physicians (means of communication, frequency, purpose, and timing) - Individuals involved in the information exchange (e.g., practice owners, staff, care taking physicians) - Decisions regarding the direct exchange of personal information versus delegation to staff - Quality of information exchange (what methods are effective with whom and at what time?) - Variations in information exchange (quality, content, and satisfaction) between practices, hospitals, and care institutions |
| Networks among physicians | - General collaboration and information exchange among physicians in the region - Communication and interactions unrelated to individual patient cases (e.g., continuing education) - Types of information shared and discussed - Structure and organization of network meetings and exchanges |
| Information exchange and pandemic | - Impact of the pandemic on patient contact, including consultation sessions and methods of information exchange (e.g., increased online meetings, use of email, postal communication, etc.) - Improvements and challenges in patient information exchange due to pandemic-related measures |
| Digital technologies used in the practice | - Types of digital technologies utilized, including their purpose, timing, use cases, target users, and methods of implementation - Level of interest in adopting digital technologies within the practice - Support requirements for integrating digital technologies into the practice - Desired improvements to existing digital tools currently in use |
