# Supplementary material for: The Role of the Installed Base in Information Exchange Among General Practitioners in Germany: Mixed Methods Study
Source: J Med Internet Res. 2025 Mar 24;27:e65241. doi: 10.2196/65241 (PMC11976167; doi:10.2196/65241)
Supplement: Multimedia Appendix 3 [file jmir_v27i1e65241_app3.docx]

| **Socio-demographic variable** | **Exchange with** | **Exchange by** | **Correlation** | ***P* value** | **95% CI low** | **95% CI high** |
| --- | --- | --- | --- | --- | --- | --- |
| **Age** | Inpatient care | Fax | -0,196 | *P*=.002 | -0.313 | -0.073 |
| **Age** | Therapists | Telephone | 0,187 | *P*=.003 | 0.064 | 0.305 |
| **Age** | Other physicians | Telephone | 0,162 | *P*=.01 | 0.038 | 0.281 |
| **Age** | Patients | E-mail | -0,161 | *P*=.01 | -0.280 | -0.037 |
| **Age** | Inpatient care | Postal mail | 0,151 | *P*=.002 | 0.027 | 0.271 |
| **Age** | Inpatient facilities | Postal mail | 0,135 | *P*=.03 | 0.010 | 0.256 |
| **Age** | Health departments | Postal mail | 0,134 | *P*=.04 | 0.009 | 0.255 |
| **Age** | PMS | Fax | -0,133 | *P*=.04 | -0.254 | -0.008 |
| **Age** | Health departments | E-mail | -0,127 | *P*=.046 | -0.248 | -0.002 |
| **Age** | Outpatient care | Fax | -0,125 | *P*=.049 | -0.246 | 0.000 |
| **SoP** | Health departments | Fax | 0.176 | *P*=.005 | 0.053 | 0.294 |
| **SoP** | Outpatient care | Telephone | -0.166 | *P*=.008 | -0.284 | -0.043 |
| **SoP** | PMS | Telephone | -0.150 | *P*=.02 | -0.269 | -0.026 |
| **SoP** | Therapists | Fax | 0.147 | *P*=.02 | 0.023 | 0.266 |
| **SoP** | Other physicians | Fax | 0.135 | *P*=.03 | 0.011 | 0.255 |
| **YoPE** | Inpatient care | Postal mail | 0.187 | *P*=.003 | 0.064 | 0.304 |
| **YoPE** | Inpatient facilities | Postal mail | 0.185 | *P*=.003 | 0.022 | 0.302 |
| **YoPE** | Therapists | Telephone | 0.175 | *P*=.005 | 0.052 | 0.293 |
| **YoPE** | Health departments | Postal mail | 0.145 | *P*=.02 | 0.021 | 0.264 |
| **YoPE** | PMS | Telephone | 0.143 | *P*=.02 | 0.019 | 0.262 |
| **YoPE** | Patients | Fax | 0.139 | *P*=.03 | 0.015 | 0.259 |
| **YoPE** | Inpatient care | Fax | -0.136 | *P*=.03 | -0.256 | -0.012 |
| **YoPE** | Other physicians | Telephone | 0.130 | *P*=.04 | 0.006 | 0.250 |
| **YoPE** | Outpatient care | Telephone | 0.129 | *P*=.04 | -0.005 | 0.249 |
