# Supplementary material for: The Role of the Installed Base in Information Exchange Among General Practitioners in Germany: Mixed Methods Study
Source: J Med Internet Res. 2025 Mar 24;27:e65241. doi: 10.2196/65241 (PMC11976167; doi:10.2196/65241)
Supplement: Multimedia Appendix 4 [file jmir_v27i1e65241_app4.docx]

| **Socio-demographic variable** | **Exchange with** | **Correlation** | ***P* value** | **95% CI low** | **95% CI high** |
| --- | --- | --- | --- | --- | --- |
| **Age** | Therapists | 0.229 | *P*<.001 | 0.108 | 0.345 |
| **Age** | Medical supply stores | 0.168 | *P*=.01 | 0.029 | 0.272 |
| **SoP** | Pharmacies | -0.148 | *P*=.03 | -0.267 | -0.025 |
| **YoPE** | Patients | 0.130 | *P*=.04 | 0.006 | 0.250 |
| **YoPE** | Health departments | 0.130 | *P*=.04 | 0.006 | 0.250 |
| **YoPE** | Inpatient facilities | 0.134 | *P*=.03 | 0.010 | 0.254 |
| **YoPE** | Outpatient care | 0.171 | *P*=.007 | 0.048 | 0.289 |
| **YoPE** | Inpatient care | 0.149 | *P*=.02 | 0.025 | 0.268 |
| **YoPE** | Therapists | 0.241 | *P*<.001 | 0.121 | 0.355 |
| **YoPE** | Medical supply stores | 0.220 | *P*<.001 | 0.099 | 0.335 |
