# Supplementary material for: The Role of the Installed Base in Information Exchange Among General Practitioners in Germany: Mixed Methods Study
Source: J Med Internet Res. 2025 Mar 24;27:e65241. doi: 10.2196/65241 (PMC11976167; doi:10.2196/65241)
Supplement: Multimedia Appendix 5 [file jmir_v27i1e65241_app5.docx]

| **Socio-demographic variable** | **Barriers of digital communication** | **Correlation** | ***P* value** | **95% CI low** | **95% CI high** |
| --- | --- | --- | --- | --- | --- |
| **Age** | Additional technical effort | 0.294 | *P*<.001 | 0.172 | 0.407 |
| **Age** | Own (GPs’) insecurity in dealing with digital technologies | 0.269 | *P*<.001 | 0.145 | 0.384 |
| **Age** | Technically immature | 0.267 | *P*<.001 | 0.144 | 0.382 |
| **Age** | Not very practical | 0.235 | *P*<.001 | 0.109 | 0.353 |
| **Age** | Additional time effort | 0.212 | *P*<.001 | 0.085 | 0.332 |
| **Age** | Lack of technical requirements of patients | 0.195 | *P*=.003 | 0.068 | 0.316 |
| **Age** | Technical incompatibility | 0.192 | *P*=.004 | 0.064 | 0.313 |
| **Age** | Lack of acceptance by patients | 0.189 | *P*=.004 | 0.061 | 0.31 |
| **Age** | Insecurity in dealing with digital technologies among patients | 0.147 | *P*=.03 | 0.019 | 0.271 |
| **Sex** | Insufficient data transfer rates | -0.240 | *P*<.001 | -0.358 | -0.114 |
| **Sex** | Own (GPs’) insecurity in dealing with digital technologies | -0.186 | *P*=.002 | -0.306 | -0.059 |
| **Sex** | Legal reservations | -0.184 | *P*=.005 | -0.306 | -0.056 |
| **Sex** | Data protection concerns | -0.132 | *P*=.04 | -0.255 | -0.004 |
| **SoP** | High cost | 0.209 | *P*<.001 | 0.083 | 0.328 |
| **SoP** | Data protection concerns | 0.189 | *P*=.004 | 0.063 | 0.309 |
| **SoP** | Insecurity in dealing with digital technologies among patients | 0.156 | *P*=.02 | 0.029 | 0.279 |
| **ToO** | High cost | 0.013 | *P*=.02 | -0.255 | -0.004 |
| **YoPE** | Technically immature | 0.256 | *P*<.001 | 0.133 | 0.371 |
| **YoPE** | Additional time effort | 0.225 | *P*<.001 | 0.099 | 0.343 |
| **YoPE** | Additional technical effort | 0.215 | *P*<.001 | 0.090 | 0.334 |
| **YoPE** | Not very practical | 0.206 | *P*<.001 | 0.080 | 0.326 |
| **YoPE** | Own (GPs’) insecurity in dealing with digital technologies | 0.203 | *P*=.002 | 0.077 | 0.322 |
| **YoPE** | Technical incompatibility | 0.202 | *P*=.002 | 0.075 | 0.323 |
| **YoPE** | Lack of technical requirements of the patients | 0.191 | *P*=.003 | 0.065 | 0.312 |
| **YoPE** | Insufficient data transfer rates | 0.171 | *P*=.009 | 0.043 | 0.294 |
| **YoPE** | High cost | 0.156 | *P*=.02 | 0.028 | 0.279 |
